# A field‐deployable insulated isothermal RT‐PCR assay for identification of influenza A (H7N9) shows good performance in the laboratory

**DOI:** 10.1111/irv.12646

**Published:** 2019-09-05

**Authors:** Ken Inui, Tung Nguyen, Hsin‐Jou Tseng, ChuanFu Mark Tsai, Yun‐Long Tsai, Simon Chung, Pawin Padungtod, Huachen Zhu, Yi Guan, Wantanee Kalpravidh, Filip Claes

**Affiliations:** ^1^ Food and Agriculture Organization of the United Nations (FAO) Hanoi Vietnam; ^2^ Department of Animal Health Hanoi Vietnam; ^3^ GeneReach USA Lexington Massachusetts; ^4^ Joint Institute of Virology (Shantou University – The University of Hong Kong) Shantou China; ^5^ State Key Laboratory of Emerging Infectious Diseases, School of Public Health The University of Hong Kong Hong Kong China; ^6^ Shenzhen Third People’s Hospital Shenzhen China; ^7^ Food and Agriculture Organization of the United Nations (FAO) Regional office for Asia and the Pacific Bangkok Thailand

**Keywords:** diagnostic, influenza A (H7N9), PCR, sensitivity, specificity

## Abstract

**Background:**

Avian influenza A (H7N9) remains circulating in China. For countries at risk of introduction of H7N9, such as Vietnam, early detection of H7N9 virus is essential for the early containment of the virus. Insulated isothermal reverse transcriptase PCR (iiRT‐PCR) is a portable PCR system that can be deployed under field conditions to identify pathogens at the sampling site. Applying PCR at the sampling site will greatly reduce the time to obtain a diagnostic result which allows the veterinary authority to take immediate action to contain disease spreading.

**Objective:**

To determine analytical and diagnostic sensitivity and specificity of the portable iiRT‐PCR for H7N9 virus detection.

**Methods:**

A panel of 59 virus isolates, including H7N9, avian influenza viruses of subtype H1 to H13, swine and human influenza viruses, Newcastle disease virus, and infectious bursal disease virus, were tested by H7 and N9 iiRT‐PCR reagents, using probes and primers specific to H7 or N9, in comparison with laboratory‐based real‐time RT‐PCR assays to determine analytical sensitivity and specificity. Fifty oropharyngeal samples from experimentally infected chicken and ducks with H7N9 and 50 non‐infected control swabs were tested by the H7 iiRT‐PCR to determine diagnostic sensitivity and specificity.

**Results:**

The H7 and N9 iiRT‐PCR reagents yielded comparable levels of analytical sensitivity and specificity with real‐time RT‐PCR for the detection of H7N9 virus. Diagnostic sensitivity and specificity of H7 iiRT‐PCR were 98% and 100%, respectively.

**Conclusion:**

The observed high sensitivity and specificity of iiRT‐PCR for H7N9 detection show its potential for early detection of H7N9 in risk‐based surveillance.

## 1 INTRODUCTION

By the end of March 2018, totally 1625 human cases were confirmed to be infected with avian influenza A (H7N9) virus in 27 provinces of mainland China.^1^ The 2016‐17 H7N9 epidemic wave [5th wave] began earlier, spread to more districts and counties in affected provinces, and had more confirmed human cases than previous epidemics. In this wave, highly pathogenic avian influenza (HPAI) was detected for the first in a human infection in Guangdong Province on February 13, 2017, with illness onset in December 2016. Thus far, 28 HPAI human infections have been identified, among which 27 reported in Guangdong, Guangxi, Hunan, Inner Mongolia, and Hebei, and 1 imported case to Taiwan [WHO, 2018]. Following the 5th wave, the Chinese Ministry of Agriculture (MOA) decided to implement nationwide H7N9 vaccination starting in September 2017. Inactivated bivalent vaccine (H5N1 Re‐8 strain + H7N9 H7‐Rel strain) replaced the previously used H5 inactivated vaccine, and vaccination was applied to all kept chicken, duck, goose and domestic quail, pigeon, and rare birds. During the 6th wave (winter season 2017‐2018), only 3 human cases and 15 animal outbreaks were reported (as of June 2018). Currently, H7N9 circulation seems to be much lower in the poultry population, with only 1 outbreak was reported in the 2018‐2019 influenza season^2^ and only one human case was recently reported in April 2019 in Jiuquan City in Gansu Province.^3^

Recent molecular characterization of H7N9 viruses showed a continuous evolution of the viruses similar to the H5N1 evolution.^4^ The latest circulating H7N9 viruses are clustered into two main lineages: the Yangtze River Delta (YRD) and Pearl River Delta (PRD) HA lineage. Additionally, certain H7N9 viruses of the YRD lineage acquired multiple basic amino acids at the cleavage site of their HA gene, converting them into highly pathogenic avian influenza (HPAI) variants. These HPAI viruses have been isolated from poultry, environmental samples, and human patients.

In neighboring countries of China (Vietnam, Lao PDR, and Myanmar), the Food and Agriculture Organization of the United Nations (FAO) has been supporting risk‐based active surveillance for influenza A and H7N9 in live bird markets and along the poultry value chain. In Vietnam, active surveillance was conducted in live bird markets (LBMs) in 26 out of 63 provinces for H5N1 and in 14 provinces for H7N9 during 2016‐2017. From April 2017 to March 2018, the surveillance has detected influenza A virus in 38 out of 107 markets sampled (22% positive at sample level) (FAO, unpublished results). These markets were located in all 26 provinces. To date, H7N9 has not been detected through surveillance activities and no human infection has been reported, suggesting the virus is not currently present in Vietnam. Based on recent risk assessments, the threat of introduction of H7N9 into Vietnam remains moderate to high based on value chain studies, which have found undocumented movement of poultry into Vietnam from China and the recent occurrence of HPAI H7N9 in poultry in China.

The current avian influenza surveillance in Vietnam relies on the testing of samples at regional animal health offices (RAHO's) or the National Centre for Veterinary Diagnosis (NCVD). The average time to obtain a laboratory result is 2.5 days, with the majority of this time being attributed to sample shipment and transport to the laboratories (6‐24 hours on average). Novel technologies, such as the insulated isothermal PCR (iiRT‐PCR), are portable PCR systems that can be applied under field conditions by the fast PCR reaction (in 42 minutes). The iiRT‐PCR portable system is a miniature portable device for field test, using freeze‐dried thermostable reagents and powered by rechargeable lithium batteries. The iiRT‐PCR applies the concept of Rayleigh‐Benard convection to drive a PCR reaction in capillary tubes. The primers/probe design rules are the same as for RT‐PCR. While conventional PCR requires multiple cycles of heating and cooling, iiRT‐PCR is performed through the creation of a temperature gradient in a capillary tube with a single heating source at the bottom of a capillary tube and establishment of thermal convection within the tube, mimicking the cycles of conventional real‐time PCR. This system has recently been developed to rapidly detect pathogenic viruses, including white spot syndrome virus, classical swine fever, foot and mouth disease, equine influenza, bluetongue virus, and MERS‐CoV.^5^, ^6^, ^7^, ^8^, ^9^, ^10^

Taking advantage of the iiRT‐PCR portable system, the aim of this study was to validate the performance of the iiRT‐PCR portable system for H7N9 detection.

## 2 MATERIALS AND METHODS

### 2.1 Viruses

This study used a panel of 59 virus isolates for analytical specificity that included 28 H7N9 AIVs, 7 H7 AIVs of Eurasian lineage but not in the cluster of recent H7N9 virus in China, 15 non‐H7 AIVs, seven non‐H7 influenza viruses of swine and human origin, two poultry viruses, Newcastle disease virus, and infectious bursal disease virus (Table 1).

**Table 1:** Viruses used in this study and the analytical specificity results of real‐time RT‐PCR and insulated isothermal RT‐PCR

Virus lineage	Isolate name	Subtype	RRT‐PCR (*C*_t_ value, 0:no* C*_t_)			iiRT‐PCR (1 pos, 0 neg)	
			M InfA	H7 CODA	H7 CNIC	H7	N9
Index virus 2013	A/Anhui/1/2013	H7N9	18.1	20.5	21.5	1	1
H7N9 viruses of Yangtze River lineage 2015‐2017 (n = 11)	A/Ck/ST/2969/2017	H7N9	16.5	18.2	19.2	1	1
	A/Ck/ST/881/2017	H7N9	19.9	19.2	19.9	1	1
	A/Ck/ST/586/2017	H7N9	15.4	17.4	17.9	1	1
	A/Ck/NJ/578/2017	H7N9	20.0	20.2	21.9	1	1
	A/Ck/HZ/656/2017	H7N9	15.5	16.6	21.0	1	1
	A/Ck/HZ/123/2017	H7N9	15.9	18.5	22.8	1	1
	A/Env/NJ/739/2017	H7N9	11.9	15.0	21.0	1	1
	A/Ck/ST/750/2017	H7N9	20.0	18.7	21.6	1	1
	A/SCk/ST/1059/2017	H7N9	13.6	16.4	20.3	1	1
	A/Ck/ST/1497/2017	H7N9	13.6	16.3	17.2	1	1
	A/Ck/ST/2439/2017	H7N9	19.4	21.8	25.9	1	1
H7N9 viruses of Pearl River lineage 2015‐2017 (n = 11)	A/Ck/GZ/1273/2017	H7N9	18.3	19.3	21.6	1	1
	A/Ck/DG/3082/2016	H7N9	17.1	18.3	20.3	1	1
	A/Ck/DG/3090/2016	H7N9	13.3	15.4	26.1	1	1
	A/Ck/DG/3097/2016	H7N9	18.9	16.7	27.4	1	1
	A/Ck/DG/A3/2017	H7N9	16.7	19.2	30.8	1	1
	A/Ck/DG/A12/2017	H7N9	13.5	16.5	28.2	1	1
	A/Ck/DG/A101/2017	H7N9	15.8	18.9	30.6	1	1
	A/Ck/GZ/361/2017	H7N9	15.8	17.1	19.5	1	1
	A/Ck/GZ/372/2017	H7N9	16.6	16.3	19.1	1	1
	A/Ck/GZ/484/2017	H7N9	21.0	20.9	21.8	1	1
	A/Ck/GZ/1183/2017	H7N9	18.4	20.1	22.3	1	1
Highly pathogenic H7N9 viruses of 2017 (n = 5)	A/ GD/17SF003/2017	H7N9	18.2	18.5	22.3	1	1
	A/Ck/GZ/418/2017	H7N9	19.6	21.6	22.7	1	1
	A/Ck/GZ/430/2017	H7N9	15.3	17.1	20.7	1	1
	A/Ck/GZ/837/2017	H7N9	13.4	16.4	17.9	1	1
	A/Ck/GZ/1494/2017	H7N9	11.8	15.2	17.3	1	1
H7 viruses of Eurasian lineage but not in the cluster of recent Chinese H7N9 (n = 7)	A/Dk/Thai/CU7280C/2010	H7N6	22.4	19.7	26.7	1	0
	A/Dk/Thai/CU7279T/2010	H7N6	18.9	18.2	22.9	1	0
	A/Dk/Thai/CU9744C/2010	H7N4	25.1	20.6	35.9	1	0
	A/Dk/Thai/CU10507T/2011	H7N4	17.6	17.6	0	1	0
	A/Dk/JX/1760/2003	H7N7	20.7	17.3	27.4	1	0
	A/Dk/VN/CT/LBM25/2013	H7	22.5	21.0	0	1	0
	A/Dk/VN/AG/LBM441/2013	H7	23.3	20.1	0	1	0
Avian influenza virus subtype H1‐H6, H8‐H13 (n = 15)	A/WB/ST/520/2000	H1N9	17.4	0	0	0	1
	A/DK/ST/1090/2000	H2N3	17.6	0	0	0	0
	A/Dk/ST/1283/2001	H3N8	16.1	0	0	0	0
	A/Dk/ST/472/2003	H4N6	18.2	0	0	0	0
	A/Ck/HK/Yu22/2002	H5N1	13.9	0	0	0	0
	A/Gs/GY/3375/2014	H5N6	14.1	0	38.1	0	0
	A/Dk/ST/22636/2008	H6N2	18.2	0	0	0	0
	A/Dk/ZZ/1883/2017	H6N6	19.6	0	0	0	0
	A/WB/HK/MP2411/2004	H8N4	16.5	0	0	0	0
	A/Quail/HK/G1/1997	H9N2	16.9	0	0	0	0
	A/Ck//WZ/598/2013	H9N2	15.3	0	0	0	0
	A/Dk/ST/1796/2001	H10N8	19.6	0	0	0	0
	A/Dk/ST/4253/2003	H11N3	14.8	0	0	0	0
	A/Mallard/VN/66MD/2004	H12N9	17.2	0	0	0	1
	A/Gull/MD/704/1977	H13N6	17.5	0	0	0	0
Swine influenza virus H1 and H3 (n = 3)	A/Swine/HK/1110/2006	H1N2	15.4	0	38.1	0	0
	A/Swine/GD/NS2788/2011	H1N1	12.8	0	0	0	0
	A/Swine/HK/NS2811/2011	H3N2	14.6	0	0	0	0
Human influenza virus H1 and H3 (n = 4)	A/California/07/2009	H1N1	17.3	0	0	0	0
	A/Michigan/45/2015	H1N1	21.6	0	0	0	0
	A/HK/1/1968	H3N2	16.7	0	0	0	0
	A/HK/4801/2014	H3N2	16.9	0	0	0	0
Newcastle disease virus (n = 1)	NCVD/A372/2012	NDV	0	0	0	0	0
Infectious bursal disease virus (n = 1)	NCVD/A102/2009	IBDV	0	0	0	0	0

### 2.2 Clinical samples

A total of 50 oropharyngeal swab samples were collected from 25 chickens and 25 ducks experimentally infected with six different strains of AIV subtype H7N9. They were confirmed to be positive for AIV H7N9 by virus isolation and used as positive samples. Another set of 50 oropharyngeal swabs were collected from the same chickens and ducks before infection. They were negative for AIV H7N9 by virus isolation and used as negative samples.

### 2.3 Nucleic acid extraction

**Figure 1:**
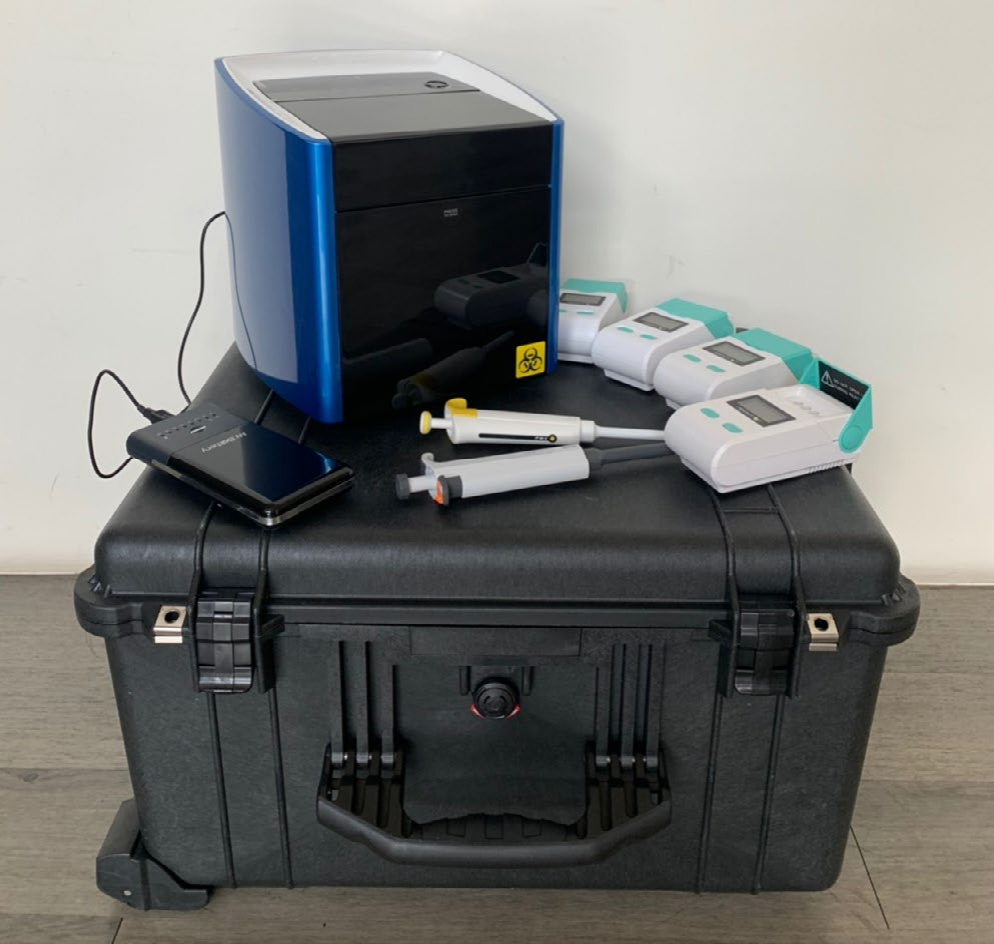
The iiRT‐PCR portable setup. This Pockit Combo set includes a Taco mini portable extractor which runs on battery or net power, four Pockit mini DNA/RNA amplifiers, a set of pipettes, and a hard case suitcase for safe transportation to the field

Nucleic acid extraction from clinical swab samples was performed with taco™ preloaded DNA/RNA Extraction Kit (GeneReach USA) on taco™ mini Nucleic Acid Automatic Extraction System (GeneReach USA) and according to the manufacturer's instructions. The Taco mini system can be driven by AC and DC current (net or battery power), and its 5 kilograms allows users to transport it to the field (Figure 1). The taco preloaded DNA/RNA  Extraction Kit is a magnetic bead‐based total nucleic extraction reagent including Lysis buffer, Washing Buffer A, Washing Buffer B, and Elution Solution are all preloaded and sealed by foil. They can be easily transported under ambient temperature and used upon needed. Briefly, 100 μL of each swab sample was added to individual wells in the first row of preloaded 48‐well extraction plate. The preloaded 48‐well extraction plate was inserted into the instrument, and the “start” button was pressed. The extraction time was 25 minutes, and the eluted nucleic acid was available in the last row of the preloaded 48‐well extraction plate. The nucleic acid was used immediately or stored at −80°C until use.

### 2.4 In vitro transcribed RNA

In vitro transcribed RNA (IVT RNA) was generated from a plasmid containing a fragment of HA gene of AIV subtype H7N9 A/Anhui/1/2013 using MAXIscript T7 kit (Ambion). Residual DNA was removed using the Ambion Turbo DNA‐free kit (Life Technologies). The concentration of RNA was measured by a NanoDrop 2000 Spectrophotometer (Thermo Fisher Scientific). Serial dilutions of in vitro transcribed RNA were made in 40 ng/μL yeast tRNA to determine the limit of detection. Single use aliquots were stored at −80°C until use.

### 2.5 POCKIT™ iiRT‐PCR assay for AIV subtype H7 and N9

POCKIT™ iiRT‐PCR assay for AIV subtype H7 was performed by using POCKIT™ influenza H7 reagent set (GeneReach USA). The N9 iiRT‐PCR was developed by using the primers and probes in the RRT‐PCR for avian influenza A (H7N9) described by WHO without any modifications.^11^ The primers and probes used are listed in Table 2. iiRT‐PCR reagent is a single dose lyophilized format which can be shipped under ambient temperature for one week.

**Table 2:** Target genes, design, and sequences of primers and probes used in this study

Platform	Assay name	Target gene and design	Sequence 5′‐3′ (F: forward, R: reverse, P: probe)		Reference
RRT‐PCR	M InfA	*M*; designed to react with most influenza A virus	F	GACCRATCCTGTCACCTCTGAC	WHO, 2017
			R	AGGGCATTYTGGACAAAKCGTCTA	
			P	FAM‐TGCAGTCCTCGCTCACTGGGCACG‐BHQ1	
	H7 CODA	*HA*; designed to react with most H7 viruses of Eurasian lineage	F	GYAGYGGYTACAAAGATGTG	OFFLU, 2013 Borm, et al 2009
			R	GAAGACAAGGCCCATTGCAA	
			P	FAM‐TGGTTTAGCTTCGGGGCATCATG‐BHQ1	
	H7 CNIC	*HA*; designed to react with Chinese H7N9 lineage but not with 100% specificity	F	AGAAATGAAATGGCTCCTGTCAA	WHO, 2017
			R	GGTTTTTTCTTGTATTTTTATATGACTTAG	
			P	FAM‐AGATAATGCTGCATTCCCGCAGATG‐BHQ1	
iiRT‐PCR	H7	*HA*; designed to react with most H7 viruses of Eurasian lineage	F	GCTTCGGGGCATCATGTT	This paper
			R	GCACCGCATGTTTCCATTCTT	
			P1	FAM‐ATTGCAATGGGCCTTGTC‐MGBNFQ	
			P2	FAM‐ATTGCAATGGGATTGGTT‐MGBNFQ	
	N9	*NA*; designed to react with most N9 viruses of Eurasian lineage	F	TAGCAATGACACACACTAGTCAAT	This paper
			R	ATTACCTGGATAAGGGTCATTACACT	
			P	FAM‐AGACAATCCCCGACCGAATGACCC‐BHQ1	

Briefly, the lyophilized reagent was reconstituted with 50 μL Premix Buffer B and mixed with 5 μL nucleic acid extract. Subsequently, 50 μL of the final mixture was transferred to an R‐tube (GeneReach USA) and sealed with a cap, spun briefly, and placed into the POCKIT™ Micro Plus Nucleic Acid Analyzer (GeneReach USA). Qualitative results were generated by the built‐in algorithm and shown on the display screen within 42 minutes. Combining nucleic acid extraction (25 minutes) with Taco mini and the iiRT‐PCR procedure (42 minutes) with POCKIT Micro Plus can generate the qualitative test results in 90 minutes.

### 2.6 Real‐time RT‐PCR (RRT‐PCR) assay for universal influenza A virus and AIV subtype H7

The M Influenza A, H7 CODA, and H7 CNIC RRT‐PCR for detection of influenza A and subtype H7 were carried out as described previously with some modifications (WHO 2017, Borm et al 2009). Primers and probes used are listed in Table 1. The Superscript III Platinum One‐Step qRT‐PCR kits (Invitrogen, Thermo Fisher Scientific) were used for all tests. The test was performed on MIC real‐time PCR platform (Bio Molecular Systems, Australia). Briefly, each 15 μL reaction mixture included 3 μL of nucleic acid sample, 7.5 μL of 2× Premix buffer, 0.8 μM of forward and reverse primers, 0.4 μM of TaqMan probe for M InfA and H7 CNIC, 0.2 μM for H7 CODA, and 0.3 μL of SuperScript III RT/Platinum *Taq* mix. The thermocycling program was set up as follows: 50°C for 30 minutes, 95°C for 2 minutes, followed by 40 cycles of 95°C for 15 seconds, and 60°C for 45 seconds. *C*_t_ thresholds were set automatically as per manufacturers’ defaults.

### 2.7 Statistical analysis

Limit of detection 95% (LOD95%) of a reaction was determined by probit analysis at 95% confidence interval by SPSS v14 (SPSS). The 2 × 2 contingency tables were analyzed by kappa statistic using SPSS to determine the inter‐assay agreement.

## 3 RESULTS

### 3.1 Analytical sensitivity of iiRT‐PCR for AIV subtype H7

The analytical sensitivity of AIV H7 iiRT‐PCR was determined by using 3‐fold serial dilutions of IVT RNA. Analysis of replicates of 100, 33, 10, and 0 copies of IVT RNA showed that 100% (20/20), 100% (20/20), 90% (18/20), and 0% (0/0), respectively, of the reactions produced positive signals. The LoD_95%_ was estimated to be 11 copies/reaction of IVT RNA by probit regression analysis. The hit rates of the AIV N9 iiRT‐PCR for 300, 100, 33, 10, and 0 were 100% (5/5), 58.33% (7/12), 57.14% (4/7), 0% (0/7), and 0% (0/12), with an estimated LoD95_95%_ of 171 copies/reaction.

The analytical sensitivity of AIV H7 iiRT‐PCR was further analyzed using 10‐fold serial dilutions (up to 10^−8^) of representative viruses from four groups of AIV H7N9 viruses. The detection endpoint (all triplicates positive) was compared to that of RRT‐PCR assays using primers and probe set of H7 CODA and H7 CNIC. The sensitivity of AIV H7 iiRT‐PCR was shown to be comparable to that of H7 CODA RRT‐PCR assay in detecting the 4 representative H7N9 viruses, while having at least one log higher sensitivity when compared to that of H7 CNIC RRT‐PCR assay (Table 3). The sensitivity of AIV N9 iiRT‐PCR was shown to be about one log lower than that of H7 CODA RRT‐PCR assay in detecting the four representative H7N9 viruses, and sensitivity similar to that of H7 CNIC RRT‐PCR assay (Table 3).

**Table 3:** Diagnostic sensitivity. Comparison of triplicate testing of ten‐fold dilutions of four avian influenza virus H7N9 strains in H7 CODA protocol and H7 CNIC protocol in real‐time RT‐PCR and in H7 and N9 insulated isothermal RT‐PCR

Virus strain	Virus dilution	RRT‐PCR (*C*_t_ value)						iiRT‐PCR (1: positive, 0: negative)					
		H7 CODA			H7 CNIC			H7			N9		
A/Anhui/1/2013 (Index virus of 2013)	−3	28.4	28.1	28.4	28.2	28.7	29.0	1	1	1	1	1	1
	−4	31.8	31.5	31.7	32.5	32.7	32.3	1	1	1	**1**	**1**	**1**
	−5	**35.6**	**35.6**	**35.1**	**34.9**	**37.6**	**37.1**	1	1	1	1	1	0
	−6	37.4	neg	neg	36.7	37.5	neg	**1**	**1**	**1**	1	1	0
	−7	neg	neg	neg	neg	neg	neg	1	0	0	0	0	0
	−8	neg	neg	neg	neg	neg	neg	0	0	0	0	0	0
A/Ck/GZ/1273/2017 (Pearl river lineage)	−3	26.0	26.0	26.3	32.7	32.9	33.1	1	1	1	1	1	1
	−4	27.1	30.3	28.9	**36.5**	**35.4**	**37.1**	1	1	1	**1**	**1**	**1**
	−5	31.5	32.9	33.4	37.4	38.5	neg	1	1	1	1	1	0
	−6	**35.6**	**36.3**	**36.8**	neg	neg	neg	**1**	**1**	**1**	1	0	0
	−7	38.0	37.3	neg	neg	neg	neg	1	1	0	0	0	0
	−8	neg	neg	neg	neg	neg	neg	0	0	0	0	0	0
A/Ck/ST/2969/2017 (Yangtze river lineage)	−3	26.0	26.8	26.3	30.7	30.2	30.9	1	1	1	1	1	1
	−4	29.2	28.9	29.0	33.1	33.1	33.6	1	1	1	1	1	1
	−5	32.9	33.2	33.0	**36.1**	**36.2**	**36.3**	1	1	1	**1**	**1**	**1**
	−6	**35.2**	**36.7**	**35.5**	neg	neg	neg	**1**	**1**	**1**	1	0	0
	−7	neg	neg	neg	neg	neg	neg	1	1	0	0	0	0
	−8	neg	neg	neg	neg	neg	neg	0	0	0	0	0	0
A/GD/17SF003/2017 (Highly pathogenic)	−3	25.6	25.6	25.7	30.8	30.9	30.6	1	1	1	1	1	1
	−4	28.9	28.9	28.8	**33.5**	**34.6**	**34.8**	1	1	1	1	1	1
	−5	32.6	32.7	32.0	37.7	37.3	neg	1	1	1	**1**	**1**	**1**
	−6	**34.5**	**35.3**	**35.7**	neg	neg	neg	**1**	**1**	**1**	0	0	0
	−7	neg	neg	neg	neg	neg	neg	0	0	0	0	0	0
	−8	neg	neg	neg	neg	neg	neg	0	0	0	0	0	0

Note

The starting titers of the viruses tested in Table 3 were 1.67E+08 and 3.78E + 08 TCID50/mL on MDCK cells for A/Anhui/1/2013 and A/GD/17SF003/2017, respectively. The exact titers for A/Ck/GZ/1273/2017 and A/Ck/ST/2969/2017 were not determined as these were fresh isolates (but considered to be around 1.0 ~ 5.0 E+08 TCID50/mL).

Abbreviation: Neg, negative.

### 3.2 Analytical specificity of iiRT‐PCR for AIV subtype H7 and N9

The analytical specificities of the AIV H7 and N9 iiRT‐PCR assay were evaluated using RNA extracted from 34 AIV H7 viruses (11 H7N9 Yangtze River lineage, 11 H7N9 Pearl River lineage, five highly pathogenic H7N9 strains, seven H7 of Eurasian lineage but not in the cluster of recent Chinese H7N9) and 24 non‐H7 viruses (15 from H1—H6 and H8—H 13, three swine influenza virus H1 and H3, four human influenza virus H1 and H3, one Newcastle disease virus, and one infectious bursal disease virus; Table 1). The assay showed 100% specificity detecting all 34 H7 viruses and showing no cross‐reactivity with non‐H7 viruses. The analytical specificity of the AIV H7 iiRT‐PCR assay was shown to be comparable to that of H7 CODA RRT‐PCR assay.

The analytical specificity of the AIV N9 iiRT‐PCR assay was evaluated similarly using RNA extracted from 29 AIV N9 viruses and 30 non‐N9 viruses. The assay showed 100% specificity, detecting all 29 N9 viruses and showing no cross‐reactivity with non‐N9 viruses.

### 3.3 Diagnostic sensitivity and specificity of AIV H7 iiRT‐PCR evaluated with oropharyngeal swabs from experimentally infected chickens and ducks

A total of 50 virus isolation‐positive and 50 virus isolation‐negative reference samples from chickens and ducks experimentally infected with different subtype H7N9 AIVs were tested by the H7 iiRT‐PCR and compared in a 2 × 2 table (Table 4). Using virus isolation as the gold standard, the iiRT‐PCR showed a sensitivity of 98% and a specificity of 100%.

**Table 4:** Diagnostic sensitivity and specificity of avian influenza virus H7 iiRT‐PCR as compared to virus isolation

	H7 iiRT‐PCR			Sensitivity	Specificity
	Positive	Negative	Total		
Virus isolation					
Positive	49	1	50	98% (49/50)	
Negative	0	50	50		100% (50/50)

## 4 DISCUSSION

To our knowledge, this is the first time that the iiRT‐PCR was validated for H7N9 detection. The validation design, including a broad set of reference viruses and the inclusion of oropharyngeal swabs of H7N9 infected birds, ensured that the analytical and diagnostic testing was performed according to the guidelines in the OIE Terrestrial Manual^12^.

Validation results show that the iiRT‐PCR system's performance is equivalent to a widely used laboratory‐based real‐time PCR setup. The analytical detection limits of the H7 and N9 iiRT‐PCR reagents are similar to RRT‐PCR, and excellent specificity to H7 and N9 viruses was observed, respectively. It should be noted that the sensitivity of the N9 protocol is about one log lower than the H7 iiRT‐PCR. A possible explanation is that the H7 primers and probes were optimized for use on the Pockit PCR, while for N9 the exact CNIC China design was used. It was shown before that also in qPCR the CNIC N9 protocol is less sensitive than various H7 protocols tested.^13^

The diagnostic performance of the H7 iiRT‐PCR with a relatively high sensitivity of 98% and a 100% specificity shows that the system would be performant to be used as a diagnostic platform. Ideally, the diagnostic performance would be tested on a larger set of samples, yet practically it showed difficult to obtain sufficient clinical samples for validation purposes. This current validation was aimed to validate the fitness of the iiRT‐PCR for the early diagnosis of H7 and N9 in animal samples (oropharyngeal swabs). Further validation is needed to show the possibility to use other sample types (environmental samples, human swabs) or to use the system to detect different HA (H5, H9) or NA‐types (N6 or N8).

Recently, other H7Nx were also found to be circulating in China and Southeast Asia. These H7Nx belong to the Eurasian H7‐lineage and are distinct from the Chinese H7N9. The current H7 test will most likely also detect these viruses as the H7 iiRT‐PCR primer/probe design targets a broad spectrum of H7. For public health purposes and early warning, this possible cross‐reactivity can be considered beneficial as any type of H7 is potentially zoonotic and detection of H7Nx in livestock samples can serve as an early warning system to raise public awareness of circulating animal influenza with pandemic potential. Also, the N9 protocol can detect other non‐H7N9 viruses, for example, H1N9 or H12N9 as shown in Table 1. Further steps in these cases should be confirmation of the subtype in the laboratory, followed by full HA and NA (or full genome) sequencing.

The innate characteristics of the iiRT‐PCR system make it a very useful tool for onsite diagnosis. The device is compact (hand‐held), uses lyophilized thermostable PCR reagents, and runs on rechargeable batteries, enabling it to be used in various field conditions (live bird markets, farms, quarantine stations, veterinary stations, road check points, or hospitals) and thus reducing time of sample transportation. Having a quick result onsite will make it possible to initiate a quick response, such as initial movement control and quarantine measures in live bird markets or on farms, reducing the risks of further spread and potential human infections of H7N9. In Vietnam for example, the portable PCR was introduced into the active live bird market surveillance for the early detection of H7N9. Once a case of H7 would be detected in the field through the portable system, immediate movement restrictions will be put in place while waiting for confirmatory diagnosis from the laboratory.

Results from field pilot studies (unpublished data) showed that it was feasible to install the system rapidly at any given sites and that personnel in the flied could be trained in the use of the iiRT‐PCR system within 2 days. One of the challenges of the system is how it will be incorporated into current ongoing active surveillance designs. iiRT‐PCR can be complementary to current surveillance designs, yet it needs to be clear if and how results will be confirmed by standard laboratory‐based tests (real‐time RT‐PCR, virus isolation, or sequencing) and how data sharing will occur from local levels to province or central levels.

In conclusion, this study showed that field‐based portable PCR (iiRT‐PCR) can be used for the early diagnosis of H7N9 as an alternative approach to laboratory‐based real‐time PCR. Using field‐based test will reduce the time to obtain a result and will enable possible quick response measures in the field, reducing the risk of further spread and human infections with H7N9 in currently non‐infected countries.
